# Course of Symptoms and Health-Related Quality of Life during Specialized Pre-Dialysis Care

**DOI:** 10.1371/journal.pone.0093069

**Published:** 2014-04-03

**Authors:** Moniek C. M. de Goeij, Gurbey Ocak, Joris I. Rotmans, Jan-Willem Eijgenraam, Friedo W. Dekker, Nynke Halbesma

**Affiliations:** 1 Department of Clinical Epidemiology, Leiden University Medical Center, Leiden, The Netherlands; 2 Department of Nephrology, Leiden University Medical Center, Leiden, The Netherlands; 3 Department of Internal Medicine, Nephrology, Groene Hart Hospital, Gouda, The Netherlands; Leiden University Medical Centre, Netherlands

## Abstract

**Background:**

Concerns are present on the limited value of renal function alone in defining the optimal moment to start dialysis. Disease-related symptoms and health-related quality of life (HRQOL) may have additional clinical value in defining this moment, but little is known about how these parameters change during pre-dialysis care. The aims of our study were to describe the course of symptoms and HRQOL during pre-dialysis care and to investigate their association with poor health outcomes.

**Methods:**

In the prospective PREPARE-2 cohort, incident patients starting specialized pre-dialysis care were included when referred to one of the 25 participating Dutch outpatient clinics (2004–2011). In the present analysis, 436 patients with data available on symptoms and HRQOL were included. Clinical data, symptoms (revised illness perception questionnaire), and HRQOL (short form-36 questionnaire; physical and mental summary score) were collected every 6-month interval. A time-dependent Cox proportional hazard model was used to associate symptoms and HRQOL with the combined poor health outcome (i.e. starting dialysis, receiving a kidney transplant, and death).

**Results:**

All symptoms increased, especially fatigue and loss of strength, and both the physical and mental summary score decreased over time, with the most pronounced change during the last 6–12 months of follow-up. Furthermore, each additional symptom (adjusted HR 1.04 (95% CI, 1.00–1.09)) and each 3-point lower physical and mental summary score (adjusted HR 1.04 (1.02–1.06) and 1.04 (1.02–1.06) respectively) were associated with a higher risk of reaching the combined poor health outcome within the subsequent 6 months.

**Conclusions:**

The number of symptoms increased and both the physical and mental HRQOL score decreased during pre-dialysis care and these changes were associated with starting dialysis, receiving a kidney transplant, and death. These results may indicate that symptoms and HRQOL are good markers for the medical condition and disease stage of pre-dialysis patients.

## Introduction

A large debate is ongoing about the optimal moment to start with dialysis.[1]–[5] The last decades there has been a trend towards starting dialysis at higher levels of renal function.[6]–[9] However, many observational studies[9]–[18] and the first randomized controlled trial, the Initiating Dialysis Early And Late (IDEAL) study [19], showed no difference in patient survival between early and late initiation of dialysis. In addition, consensus has been reached within the European Renal Best Practice guidelines on the limited value of renal function alone in the decision to start with dialysis. [4] Disease-related symptoms and a low health-related quality of life (HRQOL) are highly present in pre-dialysis patients and therefore may have an added clinical value on top of renal function in this difficult decision.

Symptoms can start appearing when renal function decreases to half of the normal function and continue to rise when renal function further decreases. [20] Furthermore, several cross-sectional studies showed that a low renal function is associated with a low HRQOL[21]–[23] and that HRQOL is a good predictor for mortality and progression to end-stage renal disease in chronic kidney disease (CKD) patients [24]. However, knowledge is lacking on the specific development of symptoms and HRQOL during pre-dialysis care. Moreover, several guidelines indicating when to start dialysis already take into account the presence of symptoms. [25], [26] However, the incorporations of these symptoms into the guidelines are not consistent and specific. Furthermore, both the IDEAL study and some observational studies showed that for the decision to start with dialysis, symptoms were at least as important as renal function. [3], [4], [19], [27] In the late start group of the IDEAL study, many patients (75.9%) started dialysis earlier than intentioned mainly because of uremia. However, no prospective data is present on which symptoms and what level of HRQOL are important for nephrologists and patients in this decision.

This lack of knowledge supports the need for a descriptive study investigating the precise course of multiple symptoms and HRQOL, both physical and mental, in pre-dialysis patients. Therefore, our first aim was to describe the course of disease-related symptoms and HRQOL during pre-dialysis care (CKD stages IV–V) and our second aim was to investigate whether disease-related symptoms and HRQOL are associated with poor health outcomes.

## Methods

### Ethics Statement

The study was reviewed and approved by the medical ethics committee of the Leiden University Medical Center. The medical ethics committee or institutional review board (as appropriate) of the other participating centers (see file S1) reviewed and approved the local feasibility of the study. All participants gave their written informed consent prior to study inclusion.

### Study Design

The PREdialysis PAtient REcord-2 (PREPARE-2) study is a prospective follow-up study of incident pre-dialysis patients treated in 25 participating Dutch nephrology outpatient clinics in The Netherlands. Of these outpatient clinics 4 were academic and 21 were peripheral. Patients were included between July 2004 and June 2011, at the start of specialized pre-dialysis care (n = 502). Patients were treated by their nephrologists in a regular scheme according to the treatment guidelines of the Dutch Federation of Nephrology [28], a Dutch guideline partly based on the K/DOQI [26] and KDIGO guidelines [29]. Patients were followed until the start of dialysis, receiving a kidney transplant, death, or censoring. Censoring was defined as: moving to an outpatient clinic not participating in the PREPARE-2 study, recovery of kidney function, refusal of further study participation, lost to follow-up or August 1, 2012 (end of follow-up), whichever came first.

### Patients

To be eligible for inclusion, patients had to be at least 18 years of age and the inclusion should take place at the moment of referral to a specialized pre-dialysis outpatient clinic. In practice, this refers to incident pre-dialysis patients with an estimated glomerular filtration rate (eGFR) of less than 20–30 ml/min/1.73 m^2^. Most of these incident patients have a progressive renal function loss. Patients with a failing kidney transplant were also eligible for inclusion if the transplantation was at least one year ago.

### Data Collection

Data on demography, biometry, primary kidney disease, comorbidities, medication use, laboratory values, symptoms, and HRQOL were collected during routine visits to pre-dialysis outpatient clinics. These visits took place at the start of specialized pre-dialysis care, at the moment of reaching one of the study endpoints (throughout text indicated as endpoint) as described previously, and every intermediate 6-month interval. Laboratory data were extracted from the electronic hospital information systems or medical records. Primary kidney disease was classified according to the codes of the European Renal Association-European Dialysis and Transplantation Association. [30] Written questionnaires used to asses symptoms and HRQOL were provided during routine visits at the start of pre-dialysis care, each 6-month interval, and at the moment of reaching an endpoint. Patients were asked to fill it in at home and return the questionnaire as soon as possible. Assistance from medical staff, a family member, or a friend was allowed.

### Measurements and Definitions

The revised illness perception questionnaire (IPQ-R) [31] was used to assess the onset of symptoms. This questionnaire has been validated [32], [33] and is widely used in the field of nephrology [34]–[36]. The following symptoms from the IPQ-R (12 out of 14 symptoms) are essentially the same as those in the commonly used kidney disease specific quality of life (KDQOL) questionnaire and can be considered as uremia- or disease-related and were therefore included in our analyses [20]; pain, nausea, breathlessness, weight loss, fatigue, stiff joints, wheeziness, headaches, upset stomach, sleep difficulties, dizziness, and loss of strength. These disease-related symptoms are often called uremic symptoms because they are highly present in patients with advanced kidney failure. The onset of these symptoms during pre-dialysis care was defined as answering ‘yes’ for the first time on the question ‘I have experienced this complaint since the beginning of my kidney disease’. HRQOL was assessed with the short form-36 (SF-36) questionnaire [37], a generic validated questionnaire consisting of 36 items that can be divided into 8 subscales. The scores on the individual items within a subscale were summed and transformed to a 0–100 scale, with higher scores indicating a better HRQOL. The 8 subscales can further be divided into two summary measures; physical summary score (consisting of the 4 subscales physical functioning, role functioning physical, bodily pain and general health) and mental summary score (consisting of the 4 subscales vitality, social functioning, role functioning emotional and mental health). Only the two summary measures (unstandardized) were used in our analyses. eGFR and creatinine clearance (CrCl) were used as indicators for renal function. GFR was estimated using the 4-variable Modification of Diet in Renal Disease (MDRD) formula. [38] CrCl was estimated with the following formula: creatinine in urine (mmol/24 h) *700/serum creatinine (μmol/l), and normalized per 1.73 m^2^ of body surface area using the formula of Du Bois and Du Bois. [39] The measurement at the moment of reaching an endpoint was defined as follows; the measurement closest to the moment of reaching an endpoint and not measured more than 6 months before this moment.

### Outcome

The outcomes for our first aim were the frequencies of all symptoms and the mean physical and mental summary score over time before reaching one of the endpoints. The outcome for our second aim was reaching the combined poor health outcome during follow-up; starting dialysis, receiving a kidney transplant, and death.

### Statistical Analyses

The patients who filled in at least one HRQOL questionnaire, including the IPQ-R and the SF-36, during pre-dialysis care (n = 436) were included. Baseline characteristics were presented as mean ± standard deviation (SD) for normally distributed variables and as median and boundaries of interquartile range (IQR) for skewed variables. Missing values at baseline and at the moment of reaching an endpoint were imputed (using 20 repetitions) with the method of multiple imputation in PASW/SPSS version 20.0. This is a recommended technique when data is missing at random, in which missing data for a patient are imputed by a value that is predicted by other known characteristics of this patient. [40], [41] All characteristics presented in Table 1, all symptoms, and the physical and mental summary score (at baseline and at the moment of reaching an endpoint), follow-up time, and the type of endpoint reached (starting dialysis, receiving a kidney transplant, death, end of follow-up (August 1, 2012), or other endpoint; moving to an outpatient clinic not participating in the PREPARE-2 study, recovery of kidney function, refusal of further study participation, or lost to follow-up) were included in this imputation model. Skewed distributed continuous variables and the follow-up time were logarithmically transformed. The method of multiple imputation was chosen because data being missing completely at random is uncommon in clinical practice and multiple imputation is still a valid method when some data is missing ‘not at random’ (related to unknown characteristics). [42] At baseline, 100% of the included patients filled in at least a part of the questionnaire and this percentage dropped to 66% at the moment of reaching an endpoint.

**Table 1:** Baseline characteristics for total population and stratified by type of endpoint.

	Total	Combined poor health outcome∧	End of follow-up/Other
	*(n = 436)*	*(n = 284)*	*(n = 152)*
Age (years)	69 (56–76)	68 (54–75)	71 (60–78)
Sex (male, %)	66	67	65
Caucasian (%)	92	93	92
BMI (kg/m^2^)*	26.7±5.1	26.8±4.9	26.4±5.6
Smokers/quitters <1 year before inclusion (%)	25	24	26
Primary kidney disease (%)			
Diabetes mellitus	13	12	15
Glomerulonephritis	14	13	15
Renal vascular disease	31	30	31
Interstitial nephropathy	7	7	7
Cystic kidney disease	12	16	5
Multisystem disease	4	4	4
Other	19	18	23
eGFR (ml/min/1.73 m^2^)†	16.9±6.1	15.2±5.5	20.1±5.8
Proteinuria (g/24 h)§	1.0 (0.3–2.2)	1.3 (0.4–2.5)	0.7 (0.2–1.4)
Urea (mmol/l)•	22.2±7.0	23.0±6.9	20.4±7.0
Creatinine clearance (ml/min/1.73 m^2^)□	19.2±7.9	17.3±7.3	23.4±7.7
Systolic blood pressure (mmHg)Δ	142±22	143±22	140±23
Hemoglobin (g/dl)**	12.3±1.5	12.2±1.4	12.6±1.6
Potassium (mmol/l)‡	4.7±0.6	4.8±0.7	4.7±0.6
Bicarbonate (mmol/l)••	23.0±4.0	22.4±3.6	24.3±4.3
Comorbidities (%)			
Cardiovascular disease□□	42	41	45
Diabetes MellitusΔΔ	26	24	30

Median (boundaries of interquartile range) is given for age and proteinuria and mean ± standard deviation is given for all other normally distributed continuous variables.

∧ Defined as starting dialysis, receiving a kidney transplant, and death.

*Available for 428 patients.

† Estimated glomerular filtration rate (eGFR) is calculated with the 4-variable Modification of Diet in Renal Disease formula and available for 379 patients.

§ Available for 225 patients.

• Available for 365 patients.

□ Creatinine clearance is estimated with the formula creatinine in urine (mmol/24 h) * 700/serum creatinine (μmol/l), normalized per 1.73 m^2^ of body surface area and available for 242 patients.

Δ Available for 432 patients.

**Available for 378 patients.

‡ Available for 377 patients.

•• Available for 223 patients.

□□ Defined as presence of a cerebrovascular accident, vascular problems, angina pectoris, myocardial infarction, or decompensatio cordis.

ΔΔ Present as primary kidney disease or comorbidity.

To describe the course of symptoms and HRQOL during pre-dialysis care, the frequency of all 12 separate symptoms, the mean (standard error of the mean, SEM) total number of symptoms and the mean (SEM) physical and mental summary score at the start of pre-dialysis care and at the moment of reaching an endpoint were calculated. A linear mixed model was used to investigate the statistical difference between these two time points, adjusted for eGFR. This model takes into account correlations between repeated measurements within the same individual. Second, the mean (SEM) total number of symptoms, physical and mental summary score, eGFR, and CrCl at the moment of reaching an endpoint and the preceding 3 time points were estimated with a linear mixed model. Moreover, on each time point a Pearson’s correlation was calculated between eGFR and CrCl and the total number of symptoms, the physical summary score and the mental summary score. Furthermore, we repeated all analyses in the subgroup of patients that started dialysis, received a kidney transplant or died (combined poor health outcome) and in the subgroup of patients that reached the end of follow-up or another type of endpoint.

A time-dependent Cox proportional hazard model was used to investigate the risk of reaching the combined poor health outcome within 6 months. Symptoms were analyzed separate and as a total number. The physical and mental summary score were analyzed in steps of 3 score points (this is considered to be a clinically relevant difference [43]). The analyses were adjusted for the potential confounders age, sex, diabetes mellitus, cardiovascular disease, and time-dependent eGFR.

To test the robustness of our results, five sensitivity analyses were performed. First, a complete case analysis was performed, which is an alternative method for handling missing data. Second, we repeated our analyses subdividing the group of patients who start with dialysis into peritoneal dialysis and hemodialysis, and subdividing the group of patients reaching another outcome into receiving a kidney transplant, death, end of follow-up, and other endpoint. Third, we performed a time-dependent Cox proportional hazard model with starting dialysis as outcome instead of the combined poor health outcome. Fourth, we repeated our imputation procedure and analyses after defining our measurement at the moment of reaching an endpoint as really measured at that moment, and not in the preceding 6 months. Fifth, we used other available measurements to define weight loss and fatigue at the moment of reaching an endpoint. Weight loss was redefined as >5% of weight lost. Fatigue was redefined as scoring below the Dutch mean normal score on the vitality subscale of the SF-36 questionnaire (score of approximately 70 [44]).

## Results

### Baseline Characteristics

In total, 502 incident pre-dialysis patients were included in the PREPARE-2 study of whom 436 patients were available for analyses. Table 1 shows that the median (IQR) age of these patients was 69 (56–76) years, 66% were male and the mean ± SD eGFR was 16.9±6.1 ml/min/1.73 m^2^. The 66 patients excluded from our analyses were younger, had a lower eGFR and CrCl, experienced more proteinuria and had a higher prevalence of diabetes mellitus.

### Description of the Course of Symptoms, HRQOL and Renal Function

In total, 284 of the 436 patients (65%) reached the combined poor health outcome (median follow-up 12.4 (IQR, 5.8–21.0) months), of whom 79% started dialysis, 11% received a kidney transplant, and 10% died. Of the 152 patients that reached another endpoint (median follow-up 30.6 (IQR, 16.1–48.3) months), 33% refused further participation, 11% had a recovered kidney function, 5% moved to another center, 2% were lost to follow-up, and 49% were still in the study. The overall eGFR and CrCl decline (n = 436) were 0.16 (95% confidence interval (CI), 0.11;0.21) and 0.21 (95% CI, 0.10;0.32) ml/min/1.73 m^2^/month respectively. The median number of filled in HRQOL questionnaires was 3 (IQR 2–4) and respectively 19%, 26%, and 55% filled in 1, 2, and 3 or more questionnaires during pre-dialysis care. Only the minority were filled in with the assistance of medical staff (1%). Furthermore, 21% were filled in with the assistance of a friend or family member and 78% were filled in without assistance. After imputation all patients had at least 2 complete questionnaires.

**Figure 1:**
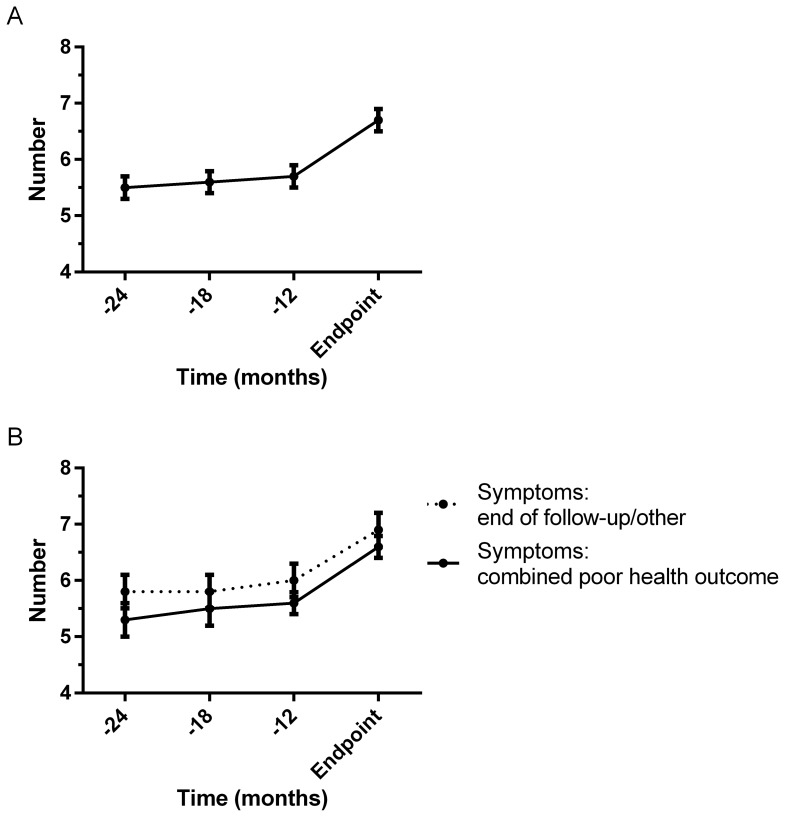
Course of symptoms during pre-dialysis care. The course of the total number of symptoms is presented for the total population (A) and stratified by the type of endpoint reached (combined poor health outcome; dialysis/transplantation/death, or end of follow-up/other endpoint, B). The black and grey dots indicate the mean and the error bars indicate the standard error of the mean (SEM). The time in months before reaching an endpoint is presented on the x-axis (−12 means that the measurement was 7–12 months before reaching an endpoint, −18 means 13–18 months etc). On the y-axis, the mean (SEM) total number of symptoms is presented, which can range from 0 to 12.

At the start of pre-dialysis care, most of the symptoms were already experienced by 20 to 40% of the patients (Table 2). Only the symptoms fatigue, stiff joints, and loss of strength were experienced by more than 50% of the patients. Furthermore, patients with diabetes mellitus or cardiovascular disease (either as primary kidney disease or comorbidity) experienced more symptoms compared to patients without (mean (SEM) total number of symptoms 4.9 (0.2) versus 4.5 (0.2)). The onset of fatigue and loss of strength were the strongest during pre-dialysis care and the onset of wheeziness, headaches, and upset stomach the lowest. The onset of all symptoms was comparable between patients reaching the combined poor health outcome (i.e. dialysis, transplantation, and death) and patients reaching another endpoint (i.e. end of follow-up and other). The mean (SEM) total number of symptoms was 4.8 (0.1) at the start of pre-dialysis care and 6.7 (0.2) at the moment of reaching an endpoint. Figure 1a depicts the course of the total number of symptoms during the last 2 years of pre-dialysis care. The onset of symptoms was most frequent during the last 6 to 12 months before reaching an endpoint, which was comparable between patients reaching the combined poor health outcome and patients reaching another endpoint (Figure 1b). On all four time points, symptoms were not correlated with eGFR or CrCl.

**Table 2:** Comparison of the presence of symptoms and HRQOL at the start of pre-dialysis care and the moment of reaching an endpoint.

	All			Combined poor health outcome∧			End of follow-up/Other		
	*(n = 436)*			*(n = 284)*			*(n = 152)*		
	Start	Endpoint	% onset§	Start	Endpoint	% onset§	Start	Endpoint	% onset§
*Symptoms*									
Pain	34.0	52.4	27.9	33.4	51.8	27.6	35.1	53.4	28.2
Nausea	29.0	46.8	25.1	30.5	45.4	21.4	26.2	49.3	31.3
Breathlessness	30.6	50.3	28.4	33.2	50.7	26.2	25.7	49.7	32.3
Weight loss	38.7	54.5	25.8	36.2	54.4	28.5	43.4	54.7	20.0
Fatigue	82.5	94.0	65.7	82.5	93.0	60.0	82.5	95.9	76.6
Stiff joints	51.3	67.9	34.1	51.5	65.4	28.7	50.8	72.7	44.5
Wheeziness	25.2	34.8	12.8	24.4	32.1	10.2	26.8	40.0	18.0
Headaches	23.0	37.1	18.3	23.3	36.1	16.7	22.4	39.1	21.5
Upset stomach	30.2	42.9	18.2	32.7	44.6	17.7	25.6	39.7	19.0
Sleep difficulties	37.9	55.3	28.0	39.6	56.3	27.6	34.7	53.5	28.8
Dizziness	33.7	50.3	25.0	34.0	47.4	20.3	33.3	55.8	33.7
Loss of strength	61.1	82.8	55.8	62.3	83.0	54.9	58.9	82.3	56.9
Number (total is 12)	4.8 (0.1)	6.7 (0.2)**	–	4.8 (0.2)	6.6 (0.2)**	–	4.7 (0.2)	6.9 (0.3)**	–
*HRQOL*									
Physical†	54.4 (1.1)	47.0 (1.3)**	–	53.5 (1.4)	44.6 (1.4)**	–	56.0 (1.9)	51.5 (2.4)	–
Mental†	67.8 (1.0)	58.9 (1.4)**	–	67.3 (1.3)	56.5 (1.5)**	–	68.9 (1.7)	63.4 (2.8)	–

Symptoms are presented as frequencies (%) and the number of symptoms and HRQOL as mean (standard error of the mean).

∧ Defined as starting dialysis, receiving a kidney transplant, and death.

§ The onset of symptoms (%) was calculated as follows: (% endpoint - % start)/(100% - % start).

† The mean physical and mental summary scores in the general Dutch population are 76.3 and 77.9 respectively.

*p<0.05, adjusted for eGFR;

**p<0.005, adjusted for eGFR, difference between baseline and the moment of reaching an endpoint, obtained with a linear mixed model.

**Figure 2:**
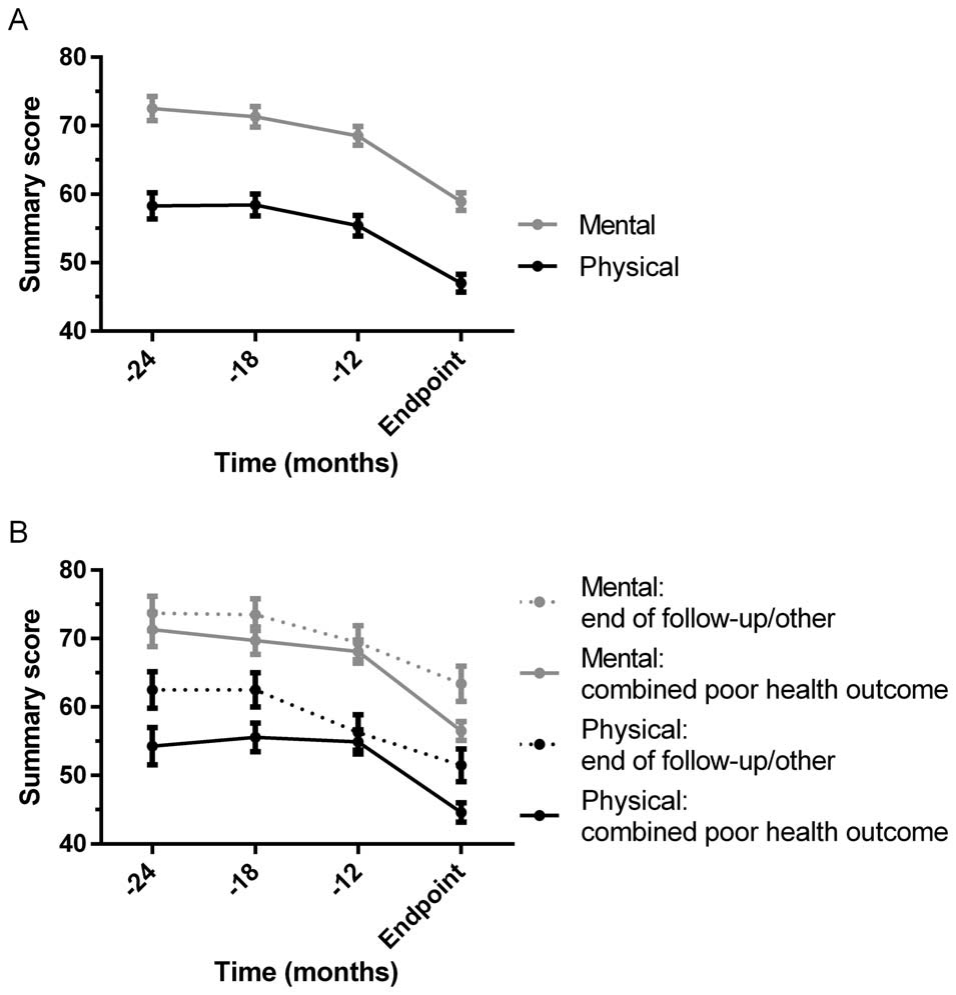
Course of HRQOL during pre-dialysis care. The course of the physical and mental summary score are presented for the total population (A) and stratified by the type of endpoint reached (combined poor health outcome; dialysis/transplantation/death, or end of follow-up/other endpoint, B). The black and grey dots indicate the mean and the error bars indicate the standard error of the mean (SEM). The time in months before reaching an endpoint is presented on the x-axis (−12 means that the measurement was 7–12 months before the moment of reaching an endpoint, −18 means 13–18 months etc). On the y-axis, the mean (SEM) score from the SF-36 questionnaire is presented (separately for the physical and mental summary measure), which can range from 0 to 100.

**Figure 3:**
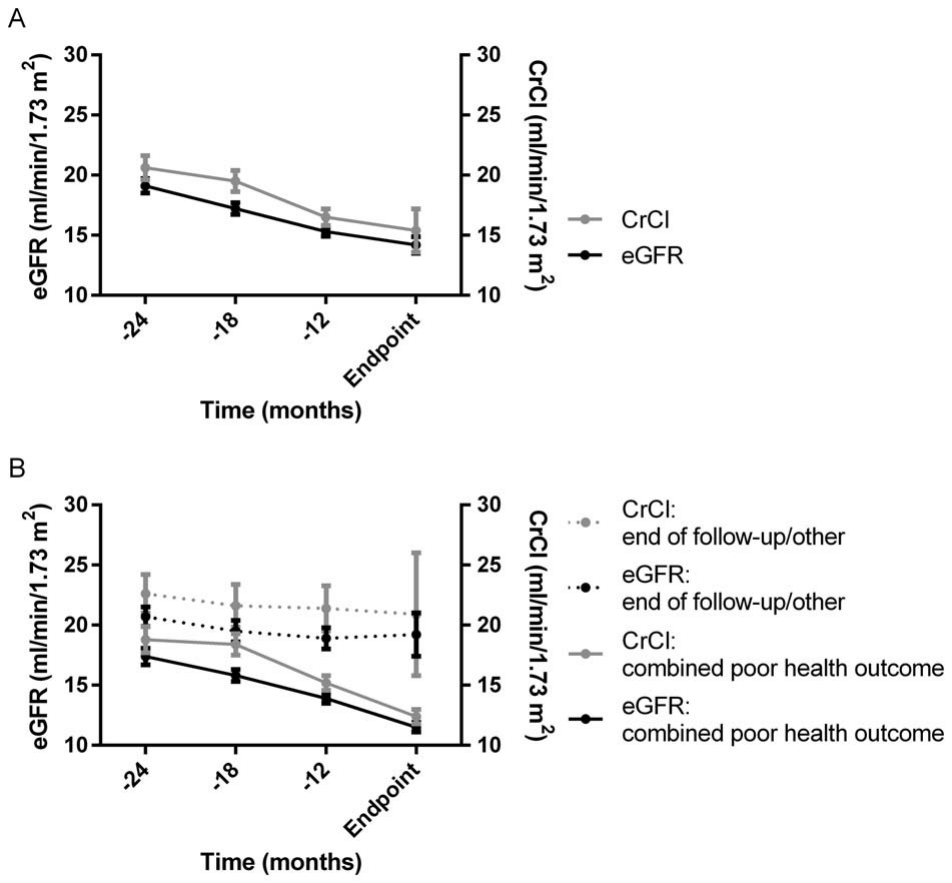
Course of eGFR and CrCl during pre-dialysis care. The course of eGFR and CrCl are presented for the total population (A) and stratified by the type of endpoint reached (combined poor health outcome; dialysis/transplantation/death, or end of follow-up/other endpoint, B). The black and grey dots indicate the mean and the error bars indicate the standard error of the mean (SEM). The time in months before reaching an endpoint is presented on the x-axis (−12 means that the measurement was 7–12 months before the moment of reaching an endpoint, −18 means 13–18 months etc). On the left y-axis, the mean (SEM) eGFR (ml/min/1.73 m^2^) is presented, and on the right y-axis the mean (SEM) CrCl (ml/min/1.73 m^2^).

The physical summary score decreased from 54.4 (1.1) at the start of pre-dialysis care to 47.0 (1.3) at the moment of reaching an endpoint (Table 2). The mental summary score decreased from 67.8 (1.0) to 58.9 (1.4). The sharpest decrease was observed during the 6 to 12 months before reaching an endpoint (Figure 2a). After stratification by the type of endpoint reached, during the last 6 months patients who started dialysis, were transplanted, or died (combined poor health outcome) showed a slightly stronger decrease of both the physical and mental summary score than patients reaching the end of follow-up or another endpoint (Figure 2b). On all four time points, no correlation was present with eGFR or CrCl. Figure 3a confirms the gradual decrease of eGFR and CrCl during pre-dialysis care, which was stronger in patients who reached the combined poor health outcome (Figure 3b).

### Association of Symptoms and HRQOL with the Combined Poor Health Outcome

The symptoms breathlessness, fatigue, wheeziness, and loss of strength showed the strongest association with reaching the combined poor health outcome (i.e. dialysis, transplantation, and death) within the subsequent 6 months (Table 3, adjusted column). Each additional symptom resulted in a crude 1.02-fold (95% CI, 0.98–1.06) increased risk, which was not statistically significant. Adjustment for potential confounders increased this HR to 1.04 (95% CI, 1.00–1.09). For HRQOL, every 3-point lower physical and mental summary score were associated with a higher risk of reaching the combined poor health outcome within the subsequent 6 months (crude HR 1.03 (95% CI 1.02–1.05) and HR 1.04 (95% CI 1.02–1.05), respectively). The results slightly increased after adjustment for potential confounders (Table 3).

**Table 3:** Association of symptoms and a low HRQOL with the risk of reaching the combined poor health outcome within the subsequent 6 months based on separate time-dependent Cox proportional hazard models.

	HR (95% CI)	Adjusted HR (95% CI)§
	*(events/n = 284/436)*	*(events/n = 284/436)*
*Symptoms*		
Pain	1.07 (0.84;1.37)	1.08 (0.84;1.40)
Nausea	1.11 (0.87;1.42)	1.06 (0.81;1.38)
Breathlessness	1.26 (0.99;1.60)	1.26 (0.98;1.62)
Weight loss	0.95 (0.74;1.22)	1.20 (0.93;1.55)
Fatigue	1.18 (0.81;1.73)	1.33 (0.90;1.97)
Stiff joints	0.97 (0.76;1.24)	1.08 (0.83;1.40)
Wheeziness	1.03 (0.79;1.34)	1.23 (0.93;1.63)
Headaches	1.02 (0.78;1.33)	1.18 (0.88;1.59)
Upset stomach	1.16 (0.91;1.49)	1.00 (0.77;1.29)
Sleep difficulties	1.17 (0.92;1.50)	1.12 (0.87;1.43)
Dizziness	0.93 (0.73;1.19)	1.06 (0.83;1.36)
Loss of strength	1.07 (0.82;1.41)	1.25 (0.95;1.65)
Number (per 1 symptom)	1.02 (0.98;1.06)	1.04 (1.00;1.09)
*HRQOL*		
Physical (per 3 score points)	1.03 (1.02;1.05)**	1.04 (1.02;1.06)**
Mental (per 3 score points)	1.04 (1.02;1.05)**	1.04 (1.02;1.06)**

The HR with its 95% confidence interval (CI) represents the increased risk of reaching the combined poor health outcome (dialysis, transplantation, and death) within the subsequent 6 months in patients with the symptom compared to patients without the symptom present, the increased risk with each additional symptom, and the increased risk with every 3-point lower physical and mental summary score. A decrease of 3 score points is considered to be clinically relevant. [43]

§ Adjusted for age, sex, diabetes mellitus, cardiovascular disease, and time dependent eGFR.

*p<0.05;

**p<0.005.

### Sensitivity Analyses

All sensitivity analyses - 1.) a complete case analysis, 2.) extensive stratification of the endpoints reached, 3.) using the start of dialysis as outcome (Table 4), 4.) defining the measurement at the moment of reaching an endpoint as really measured at that moment (not in the preceding 6 months), and 5.) redefining fatigue and weight loss - resulted in comparable courses of symptoms and HRQOL over time and similar risk estimates, implying robust results.

**Table 4:** Association of symptoms and a low HRQOL with the risk of starting dialysis within the subsequent 6 months based on separate time-dependent Cox proportional hazard models.

	HR (95% CI)	Adjusted HR (95% CI)§
	*(events/n = 225/436)*	*(events/n = 225/436)*
*Symptoms*		
Pain	1.02 (0.77;1.35)	1.04 (0.78;1.39)
Nausea	1.13 (0.85;1.49)	1.10 (0.81;1.49)
Breathlessness	1.29 (0.98;1.69)	1.28 (0.96;1.71)
Weight loss	1.00 (0.75;1.32)	1.33 (0.99;1.77)
Fatigue	1.25 (0.81;1.94)	1.48 (0.94;2.31)
Stiff joints	0.95 (0.72;1.26)	1.07 (0.80;1.44)
Wheeziness	1.06 (0.79;1.42)	1.29 (0.94;1.78)
Headaches	0.86 (0.63;1.18)	1.08 (0.76;1.53)
Upset stomach	1.08 (0.82;1.43)	0.90 (0.67;1.22)
Sleep difficulties	1.22 (0.92;1.60)	1.17 (0.89;1.55)
Dizziness	0.95 (0.72;1.26)	1.13 (0.86;1.49)
Loss of strength	1.00 (0.74;1.34)	1.20 (0.89;1.64)
Number (per 1 symptom)	1.02 (0.97;1.07)	1.05 (1.00;1.10)
*HRQOL*		
Physical (per 3 score points)	1.03 (1.01;1.05)**	1.04 (1.02;1.06)**
Mental (per 3 score points)	1.04 (1.02;1.06)**	1.04 (1.02;1.07)**

The HR with its 95% confidence interval (CI) represents the increased risk of starting dialysis within the subsequent 6 months in patients with the symptom compared to patients without the symptom present, the increased risk with each additional symptom, and the increased risk with every 3-point lower physical and mental summary score. A decrease of 3 score points is considered to be clinically relevant. [43].

§ Adjusted for age, sex, diabetes mellitus, cardiovascular disease, and time dependent eGFR.

*p<0.05;

**p<0.005.

## Discussion

In our cohort of incident patients on pre-dialysis care, many patients had already experienced symptoms at the start of pre-dialysis care. In addition, the presence of all reported symptoms increased during pre-dialysis care, especially fatigue and loss of strength. HRQOL, defined as the physical and mental summary score, decreased during pre-dialysis care. The sharpest increase of symptoms and decrease of HRQOL was observed during the last 6 to 12 months of pre-dialysis care. These results were supported by the observation that patients who reported a high number of symptoms or a low HRQOL started dialysis, received a kidney transplant, or died (combined poor health outcome) earlier than patients who reported a low number of symptoms or a high HRQOL.

To our knowledge, this is the first study to report the onset of symptoms over time in patients on specialized pre-dialysis care. Our finding that HRQOL, both physical and mental, decreased during pre-dialysis care is in line with other studies.[21]–[23] These cross-sectional studies demonstrated that HRQOL was highest in the ‘healthy’ general population, lowest in patients on dialysis, and in between for patients with CKD not on dialysis. However, the results of these studies were not CKD-stage specific and because of the cross-sectional character, the change of HRQOL over time could not be described or associated with the start of dialysis or other poor health outcomes.

Therefore, the major strength of our study is the longitudinal character, instead of a cross-sectional design. This gave us the opportunity to report the course of symptoms and HRQOL over the last years of pre-dialysis care. However, an issue that deserves further attention is that not all consecutive patients starting pre-dialysis care were included in the PREPARE-2 study, only those who were asked and willing to participate, which could have led to selection bias. Unfortunately, it is very difficult to ascertain the direction of this bias. Besides this important issue, some patients reached another endpoint than dialysis, leading to a large diversity of patients at the moment of reaching an endpoint. However, results were comparable between all type of endpoints reached. Another issue that deserves attention is that symptoms were obtained from the IPQ-R. This questionnaire is not primarily designed to assess the longitudinal presence of symptoms and does not include questions about severity. This means that the ‘yes’ category of the IPQ-R consists of patients having experienced the onset of that symptom a few years ago or just recently. This mixture of onset times in our study could have led to an underestimation of the real risks of reaching the combined poor health outcome. Besides this, symptom burden is a reliable measure, because it shows similar correlations with HRQOL and illness perceptions as symptom severity. [45], [46] This might be an indication that once the onset of a symptom has occurred (this onset coincides with a certain threshold of symptom severity) severity has no added value on a persons’ mental and physical well-being. Moreover, in answering symptom-related questions in the IPQ-R, patients’ perceptions of illness play a major role. [47] The formulation of the question may also influence the response of patients. However, more objective measures of weight loss and fatigue resulted in similar frequencies. Finally, a filled in IPQ-R and SF-36 questionnaire were not available for 66 patients. Therefore, our choice to only analyze patients with at least one filled in questionnaire could have led to selection bias. [41] However, the 66 excluded patients had a slightly worse prognosis than the 436 included patients, so our results may be an underestimation.

Uremia is defined as all the signs and symptoms accompanying advanced kidney failure that cannot be attributed to comorbid disease. The onset of uremia-related signs and symptoms can become present when renal function decreases to half of the normal function (normal eGFR is 100–120 ml/min/1.73 m^2^ on the age of 30) and continue to rise when renal function further decreases. [20] This means that uremia-related symptoms mainly become present in patients with CKD stages IV–V. Therefore, the results from our pre-dialysis cohort, showing an increase in symptoms over time and as a consequence a decrease in perceived physical and mental HRQOL, are biologically plausible. Breathlessness, weight loss, fatigue, and wheeziness were the symptoms most strongly associated with the start of dialysis (Table 4). This finding could indicate that these symptoms were considered the most by nephrologists and patients in the decision to start with dialysis. However, in our cohort data is lacking on whether the decision to start was indeed based on these symptoms. The possible role of breathlessness and wheeziness in the decision to start with dialysis could be explained by the presence of fluid overload. Fluid overload is a main indication to start with dialysis, as is stated in several guidelines [25], [26], and breathlessness and wheeziness can be consequences of pulmonary edema.

In conclusion, the presence of all symptoms increase and both physical and mental HRQOL decrease during pre-dialysis care with the sharpest change during the last 6 to 12 months before reaching an endpoint. These descriptive results may indicate that symptoms and both physical and mental HRQOL are good markers for the medical condition and disease stage of pre-dialysis patients, and are therefore good candidates to be used in defining the optimal moment to start with dialysis (or to identify those patients at a high risk of dying). Indeed we found that several symptoms and a lower level of HRQOL were already associated with the start of dialysis, which may indicate that these symptoms were already considered by nephrologists and patients in this decision. However, for each individual we need to know the exact reason for starting dialysis and the survival time on dialysis, to investigate on which specific symptoms and what level of HRQOL this decision should be based. Therefore, in the future, more research should focus on finding clinical signs and symptoms that can help to plan when to start dialysis to increase survival and HRQOL on dialysis. The European EQUAL study [48] will focus on this specific question. Eventually, a clinical decision rule based on all known factors may be a good and helpful tool for nephrologists and patients to make a more evidence based choice when a patient needs to start with dialysis.

## Supporting Information

File S1 **Supporting information file S1 contains additional information on the ethics statement including a list of the medical ethics committees or institutional review boards of all participating centers.**
(DOC)
