# Supplementary material for: Course of Symptoms and Health-Related Quality of Life during Specialized Pre-Dialysis Care
Source: PLoS One. 2014 Apr 3;9(4):e93069. doi: 10.1371/journal.pone.0093069 (PMC3974712; doi:10.1371/journal.pone.0093069)
Supplement: File S1 — Supporting information file S1 contains additional information on the ethics statement including a list of the medical ethics committees or institutional review boards of all participating centers. (DOC) [file pone.0093069.s001.doc]

**Supporting Information File S1: Ethics Statement**

The PREPARE-2 study was evaluated and approved by the medical ethics committee of the Leiden University Medical Center (LUMC). The medical ethics committee or institutional review board (as appropriate) of the other participating centers only evaluated the local feasibility of the study and thereafter gave their approval. These centers are:

- Amsterdam, Academic Medical Center
- Amsterdam, Sint Lucas-Andreas Hospital
- Amsterdam, VU Medical Center
- Apeldoorn, Gelre Hospitals
- Breda, Amphia Hospital
- Delft, Reinier de Graaf
- Den Bosch, Jeroen Bosch Hospital
- The Hague: Medical Center Haaglanden
- Ede, Hospital Gelderse Vallei
- Eindhoven, Catharina Hospital
- Emmen, Scheper Hospital (approval also included Beilen, Dialysis Clinic North)
- Goes, Admiraal de Ruyter Hospital
- Gouda, Groene Hart Hospital
- Groningen, University Medical Center Groningen
- Haarlem, Kennemer Gasthuis
- Leeuwarden, Medical Center Leeuwarden
- Leiden, Leiden University Medical Center
- Leiderdorp, Rijnland Hospital
- Roermond, Laurentius Hospital
- Roosendaal, Franciscus Hospital
- Rotterdam, Franciscus Gasthuis
- Veldhoven, Máxima Medical Center
- Zaandam, Zaans Medical Center
- Zwolle, Isala Clinics
